# Effects of aspect ratios and vertical loads on in-plane seismic behavior of unreinforced masonry walls: A numerical simulation

**DOI:** 10.1371/journal.pone.0282430

**Published:** 2023-03-02

**Authors:** Yukun Hu, Pengfei Ma, Jitao Yao

**Affiliations:** 1 School of Civil Engineering, Xi’an University of Architecture and Technology, Xi’an, China; 2 Key Lab of Structural Engineering and Earthquake Resistance, Ministry of Education, Xi’an, China; National University of Sciences and Technology, PAKISTAN

## Abstract

The in-plane seismic behavior of unreinforced masonry (URM) structures is closely related to the aspect ratio of the wall and vertical load. The purpose of this study was to investigate the difference between the failure mode of the model and the horizontal load using the finite element model (FEM) under the action of aspect ratio (0.50 to 2.00) and vertical load (0.2 MPa to 0.70 MPa). The overall macro model was established using the Abaqus software, and the corresponding simulation was performed. The simulation results indicate that: i) the shear failure and flexural failure were the main failure modes of masonry walls; ii) shear failure could be viewed as the main failure mode of the model when the aspect ratio was less than 1.00; however, the flexural failure was considered to be the main failure mode of the model once the aspect ratio was greater than 1.00; iii) when a vertical load of 0.20 MPa was applied to the model, only flexural failure was observed, regardless of whether the aspect ratio of the model increased or decreased; the flexural shear mixed failure was captured within the range of 0.30 MPa– 0.50 MPa; the shear failure was the main failure mode within the range of 0.60 MPa– 0.70 MPa; and iv) the wall with an aspect ratio less than 1.00 could bear a higher horizontal load, and the increase in vertical load can significantly improve the horizontal load of the wall. In contrast, once the aspect ratio of the wall reaches or exceeds 1.00, the increase in the vertical load has little effect on the increase in the horizontal load of the wall.

## Introduction

Brick masonry is one of the oldest and most extensively used construction forms. Brick units and mortar form different masonry walls depending on their geometric shapes, arrangements, and mechanical properties [1, 2]. The high degree of vulnerability observed in existing unreinforced masonry (URM) structures during construction is likely caused by improper seismic fortification requirements [1]. Moderate to high earthquakes caused severe partial damage and overall collapse of the URM buildings because of cracking, which caused huge economic losses and a large number of casualties [3, 4].

As the main load-bearing members of URM structures, the compressive capacity of the block is significantly better than that of tension, shear, and bending. When walls are subjected to complex external actions, macrocracks are vulnerable to emerge in URM structures, and damage and failure occur successively. By summarizing the post–earthquake failure forms of various URM structures, it can be concluded that URM walls often present horizontal, vertical, and diagonal cracks when subjected to earthquake action [5]. Furthermore, in terms of the in–plane behavior, wall failure can usually be controlled by the shear or bending of axial forces [6]. The former is mainly characterized by diagonal cracks, whereas the latter is characterized by horizontal cracks at the bottom.

Several scholars have conducted a series of studies on the different factors influencing the failure mode of masonry walls [7–12]. The main influencing factors include constructional columns and circular beams, number of floors of the building, relative stiffness ratio of the pier and spandrel, overall aspect ratio, and structural layout plan (bearing horizontal walls, longitudinal walls, longitudinal and horizontal walls, and inner frames) [9]. Different types of structural measures for masonry structures have a significant impact on the failure mode under earthquake action. Generally, a reasonable design of circular beams and constructional columns in masonry structures is regarded as an effective structural measure. When the number of floors of the masonry buildings increases by one floor, the vertical load of the wall increases by approximately 0.10 MPa [1]. Under the action of an earthquake, an increase in the vertical load leads to an increase in the shear capacity of the pier; therefore, the vertical load of the pier has a significant impact on the overall failure mode of masonry structures. In terms of the overall aspect ratio of the wall, it is easier for lower-type walls to achieve overall shear failure. In contrast, higher walls are more likely to exhibit overall flexural failure. In addition, the influence of material strength (brick and mortar) on the overall structure is also distinct. Masonry buildings with high–strength materials tend to have a higher resistance to earthquakes than masonry buildings with low–strength materials [9].

Currently, numerous experimental studies on the retrofitting and repair of masonry walls have been performed to investigate the efficiency of different reinforcement materials and the similarities and differences between different reinforcement methods [13]. As an external reinforcement method, steel ties [14] and steel strips [15] have the advantages of simple and moisture–free operation. Meanwhile, the reinforced wall not only has a higher shear strength and deformation capacity, but the failure mode of the wall can also be altered from shear sliding to toe compression. Fiber-reinforced polymer (FRP), as a relatively popular reinforcement material, can form a firm adhesion between FRP and masonry substrates using an organic epoxy resin [16–26]. Although the participation of epoxy resin stabilizes the transmission performance of shear load on the surface of masonry substrate [16–18, 24], various disadvantages of epoxy resin are also exposed in the use process [19–22]: (i) the harmfulness of epoxy resin to operators; (ii) the incompatibility of the wet masonry substrate surface; (iii) weak vapor permeability of the organic matrix; (iv) limited operability under low temperature conditions; (v) weak fire resistance of composites; (vi) inability of epoxy resin to separate from the masonry substrate (irreversibility); (vii) relatively high cost; and (viii) high requirements for surface flatness of the masonry substrate.

Accordingly, replacing the organic matrix with an inorganic matrix, that is, replacing FRP with textile-reinforced mortar (TRM) and fabric-reinforced cementitious matrix (FRCM), provides a feasible alternative to the limitations of the above epoxy resin, particularly for memorial masonry structures, which have higher requirements for the invasiveness of reinforcement materials and compatibility between materials and substrates [4, 27]. TRM and FRCM have the same advantages as FRP, such as a higher strength-to-weight ratio, relatively fast and easy operation, and versatility, which makes FRM and FRCM effective, practical, and cost–effective solutions for structural upgrading, retrofitting, and seismic reinforcement [28–31].

In the past two decades, a large number of tests on URM walls have been performed [32–39], which are primarily characterized by the comparison and analysis of the seismic performance of the walls before and after reinforcement. Undeniably, the seismic performance of the reinforced wall has been significantly improved compared to that of the original wall in every published article. However, the number of specimens in the test design is often only a few to a dozen owing to construction site restrictions and construction cost requirements. These conditions lead to the failure to comprehensively discuss some influencing factors in the analysis of experimental results, such as the in–plane performance response of walls with a specific aspect ratio under different vertical loads or the in–plane performance response of walls with different aspect ratios under a specific vertical load.

Numerical simulations provide a feasible alternative method for experimental research on the seismic performance of masonry walls [40–43], which can simulate the linear and nonlinear behaviors of masonry. In addition, numerical simulation depends on the required level of accuracy and model size and can follow either micro modeling (usually used to simulate small components) or macro modeling methods (usually used to simulate structural performance) [44, 45]. Accordingly, a series of numerical simulation studies on masonry walls was conducted to verify the compliance of the test and simulation results [46–48].

Against this background, the influence of the aspect ratio of the wall and the vertical load on the seismic performance of the masonry wall was studied using a numerical simulation method to comprehensively grasp the similarities and differences in the failure mode and lateral load of the wall under the action of different influencing factors. The remainder of this paper is organized as follows. Section 2 introduces the simulation tool and method and cites one specific test specimen to verify the effectiveness and reliability of the simulation method used in this study. Section 3 explains the specific details of the model design, load actions, constraints, and interactions. In Section 4, a total of 30 finite element models (FEMs) with different aspect ratios and vertical loads are established, the corresponding failure modes and force–displacement curves of the models are compared and discussed comprehensively, and the similarities and differences in the seismic performance of the models under different influencing factors (aspect ratio and vertical load of the masonry wall) are discussed, such as failure mode and force–displacement curve. Finally, some clear conclusions are drawn, and the next work to be carried out was put forward.

## Selection of simulation method and validation

It is well known that the reliability of numerical simulation results is closely related to the choice of the modeling method. Generally, micro models are used to simulate small components, whereas macro models are used to simulate the structural performance [44, 45]. This study aimed to discuss the similarities and differences in the failure mode and horizontal load of URM walls with different aspect ratios and vertical loads. Therefore, the ABAQUS software was adopted in this study, and a macro model was established.

**Fig 1:**
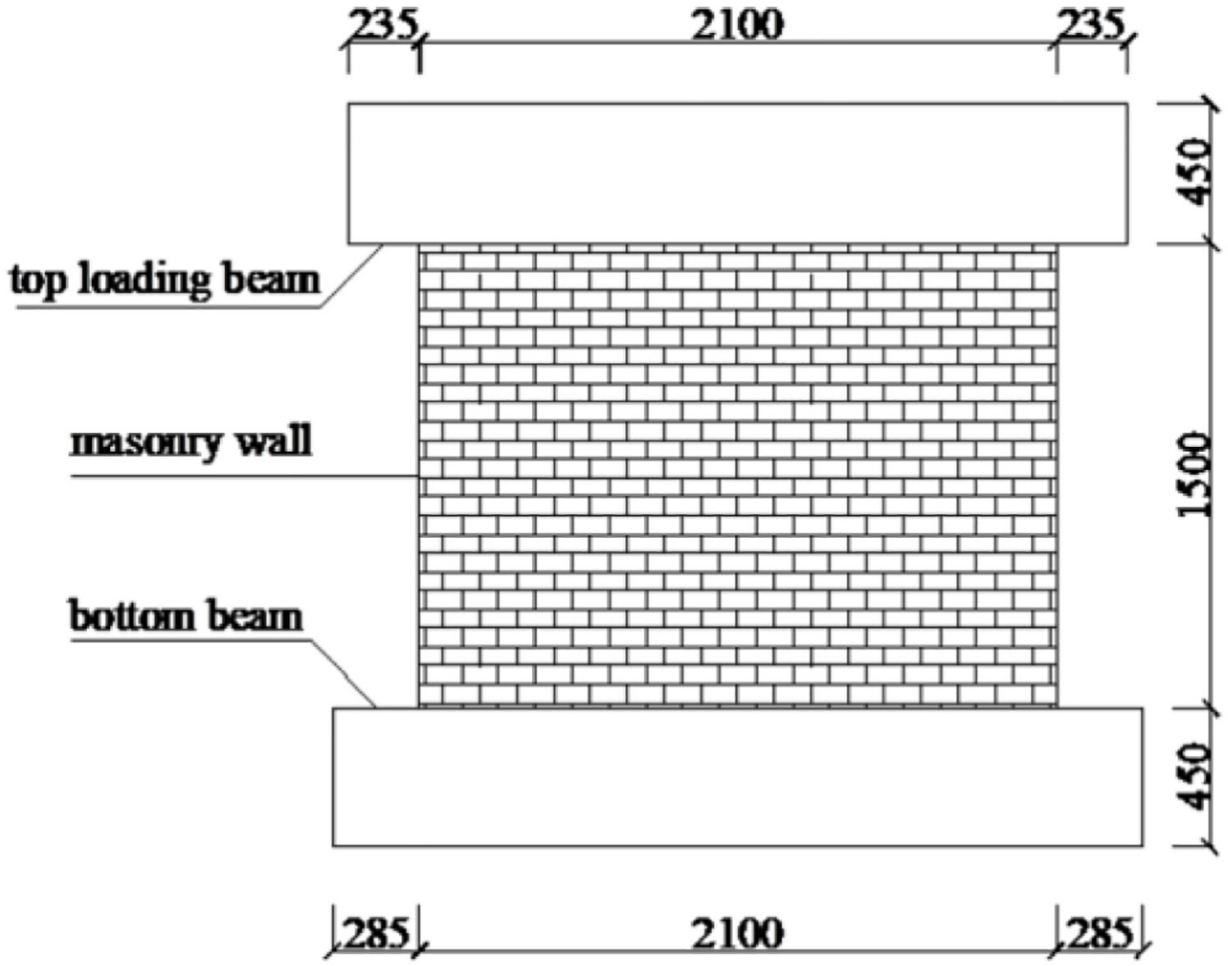
Dimensions of the specimen (mm) (reannotated based on ([16])).

In this section, the type of brick is consistent with that used in traditional existing masonry walls [22, 49–51], and the corresponding material mechanical parameters can be close to those used in most studies [22, 50–52] to ensure the universality and applicability of the selected URM wall model, and a summary of the corresponding material mechanical parameters in literature is shown in Table 1. Furthermore, to verify the workability of the selected simulation software and reliability of the simulation results, the specimen design parameters in the literature [22] were adopted (Fig 1), and the corresponding test results were compared with the simulation results obtained in this section.

**Table 1:** Summary of mechanical properties of materials in literature.

Literature	Compressive strength of brick	Compressive strength of mortar
[22]	12.80 MPa	4.80 MPa
[50]	7.50 MPa	1.80 MPa
[51]	13.00 MPa	3.20 MPa
[52]	20.00 MPa	9.60 MPa
[53]	11.30 MPa	2.40 MPa

### Mechanical parameters

**Fig 2:**
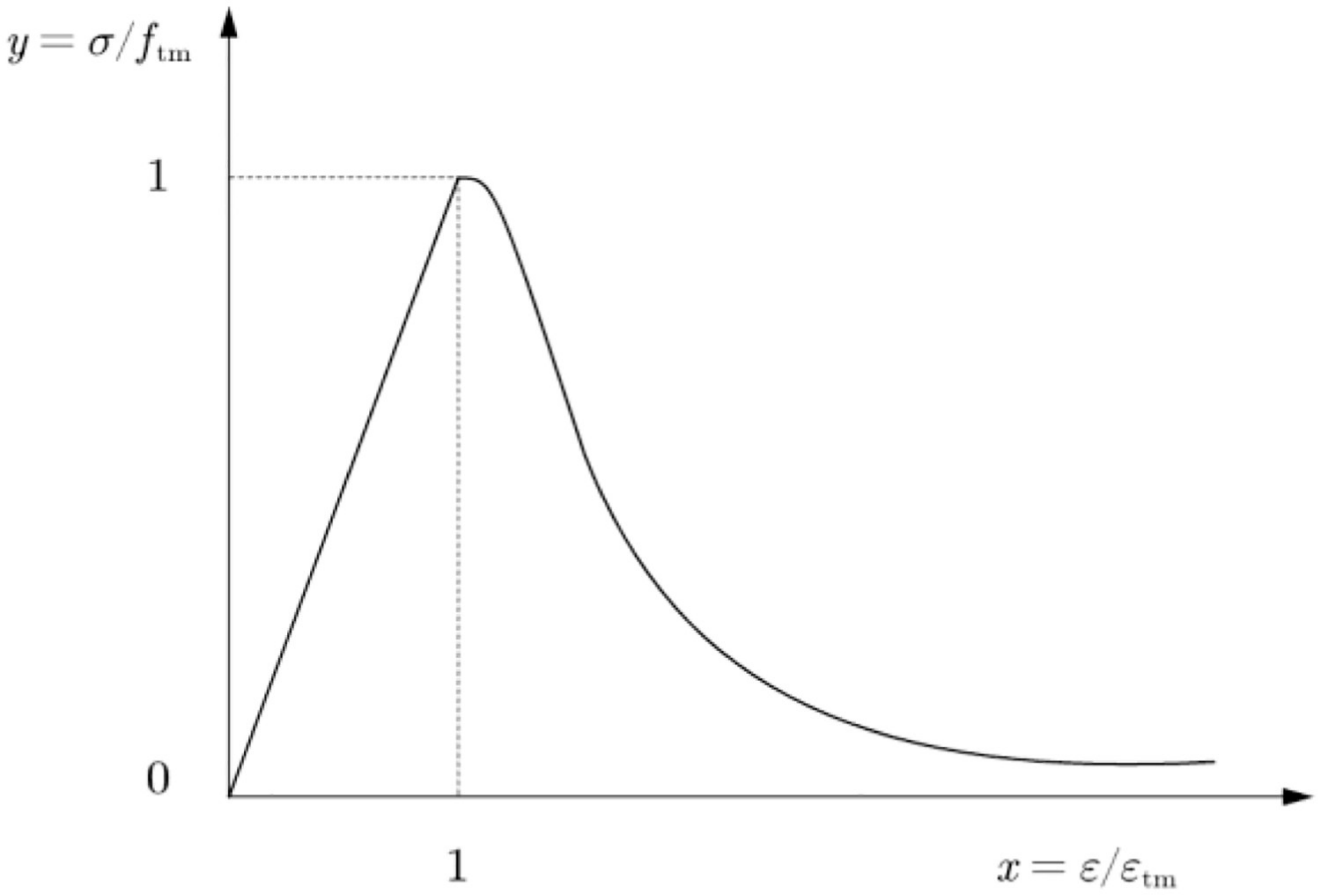
Stress-strain curve of brick masonry under uniaxial tension.

The uniaxial compression constitutive of brick masonry is given by Eq (1) [53]. *σ* and *ε* express the values of stress and strain of brick masonry, respectively; *f*_m_ expresses the stress value of the compressive stress–strain curve of brick masonry at the peak point; *ε*_m_ expresses the strain value of the corresponding point, and the value of *η* is 1.633. The tensile constitutive model of concrete was used to replace the tensile constitutive model of masonry, as expressed in Eqs (2) and (3) [9, 54]. The stress–strain curve of the brick masonry under uniaxial tension is shown in Fig 2. *f*_tm_ expresses the stress value of the tensile stress–strain relationship of the brick masonry at the peak point, and *ε*_tm_ expresses the strain value of the corresponding point.


$$\frac{\sigma }{f_{\text{m}}}\;=\frac{\eta }{1+(\eta -1){\left(\frac{\varepsilon }{\varepsilon_{\text{m}}}\right)}^{\frac{\eta }{\eta -1}}}\frac{\varepsilon }{\varepsilon_{{\text{m}}^{\prime }}}$$ (1)

$$\frac{\sigma }{f_{\text{tm}}}=\frac{\varepsilon }{\varepsilon_{\text{tm}}},x\leq 1$$ (2)

$$\frac{\sigma }{f_{\text{tm}}}=\frac{\frac{\varepsilon }{\varepsilon_{\text{tm}}}}{2{\left(\frac{\varepsilon }{\varepsilon_{\text{tm}}}-1\right)}^{1.7}+\frac{\varepsilon }{\varepsilon_{\text{tm}}}},x>1$$ (3)

The key parameters input into the concrete plastic damage (CPD model) are as follows: expansion angle = 30, eccentricity = 0.10, *f*_b0_/*f*_c0_ = 1.16, *K* = 0.6667, and viscosity coefficient = 0.005. The average compressive strength of the bricks was obtained by testing ten samples, and the final value was 12.80 MPa (COV = 0.19). The mean compressive strength of the brick masonry prisms, 2.20 MPa (COV = 0.14) [22], was obtained from compression tests conducted on six prisms. In addition, the main parameters obtained from testing brick masonry were as follows: density = 2248 kg/m^3^, elastic modulus (*E*) = 2400 MPa, and Poisson’s ratio = 0.149.

### Numerical model development

**Fig 3:**
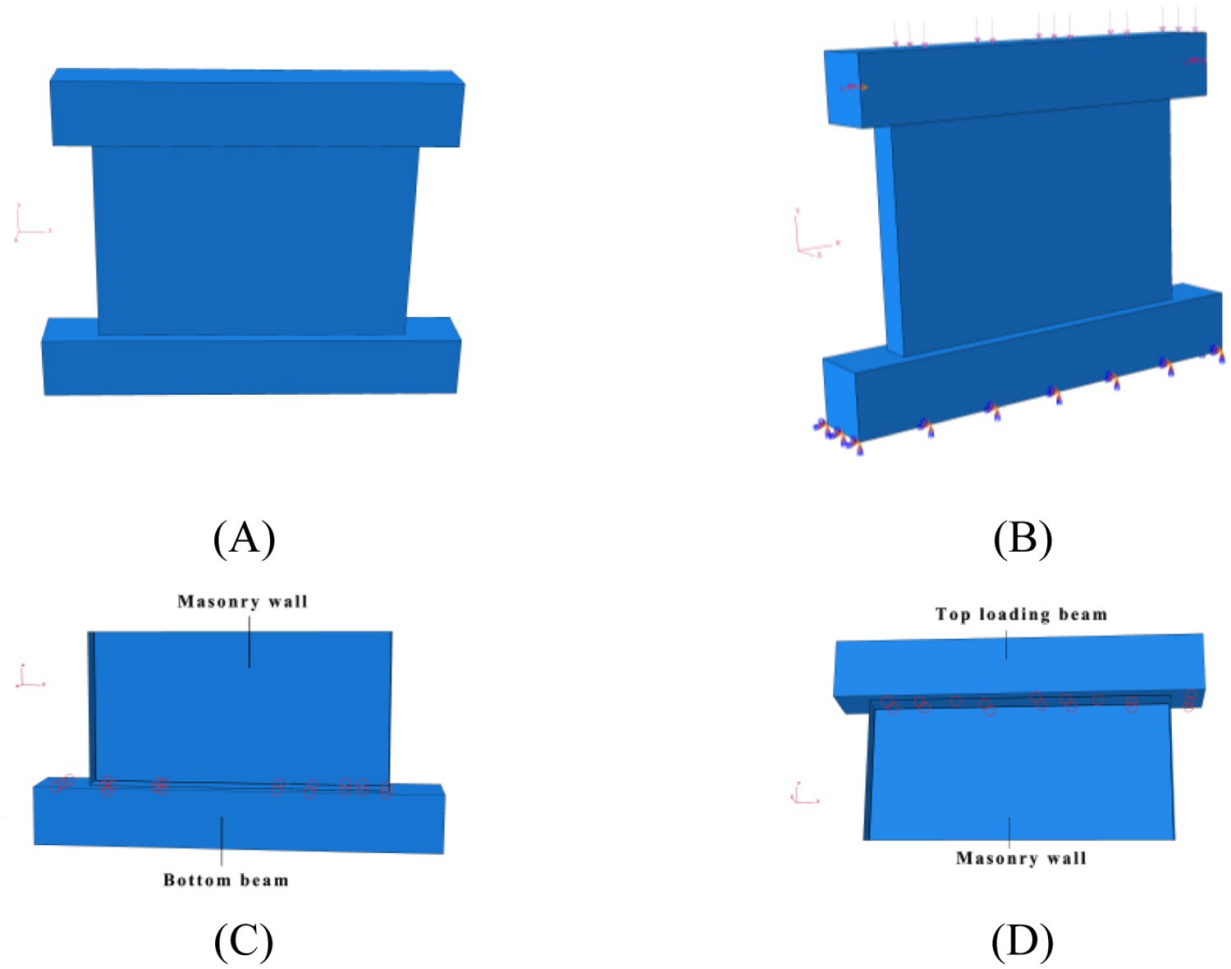
Details of finite element model. (A) Model assembly. (B) Loads and boundaries. (C) “Tie” connection between bottom beam and masonry wall. (D) “Tie” connection between top loading beam and masonry wall.

The main steps of modeling are as follows: (i) Using the integral modeling method, URM masonry wall model characterized by the dimensions 2100 mm × 1500 mm × 240 mm was established, and the aspect ratio of the corresponding model was 0.71; (ii) the model was assumed to be an isotropic uniform continuum, and the mesh size of model was set to 20 mm; (iii) the connection between each part of the model was simplified by "tie" connection; The model was formed by the assembly of the top loading beam, main masonry wall, and bottom beam; (iv) The "tie" connection method was adopted between the top loading beam, main masonry wall, and bottom beam; the boundary conditions of the bottom beam were completely fixed; (v) the model adopted two loading steps to apply the required load. First, a vertical load (0.60 MPa) was applied to the top loading beam, which was uniformly transmitted to the masonry wall by the loading beam. Subsequently, a cyclic horizontal load was applied along the horizontal direction of the loading beam. The details of the model are shown in Fig 3.

The typical pseudostatic loading protocol is consistent with that adopted in [22, 55], which consisted of a series of load- and displacement-controlled cycles. The load-controlled amplitude increment was 10 kN, starting with a load of 10 kN. A single cycle was performed at each loading stage until the first visible crack emerged. The displacement-controlled amplitude increment was 1 mm, starting with a displacement of 2 mm. Three cycles were performed during each displacement stage. The displacement rate was set to 0.05 mm/s until the end of loading. Two vertical hydraulic jacks were suspended under the reaction frame to apply vertical loads until specimen failure. The test was terminated when the seismic shear force was decreased to 85% of the maximum load.

During the simulation, the loading protocol was consistent with the above description. The load-controlled amplitude increment was 10 kN, starting with a load of 10 kN and ending with a load of 60 kN (approximately equal to the load at the first crack in the loading process). The displacement-controlled amplitude increment was 1 mm, starting with a displacement of 2 mm and ending with failure displacement.

### Validation

**Fig 4:**
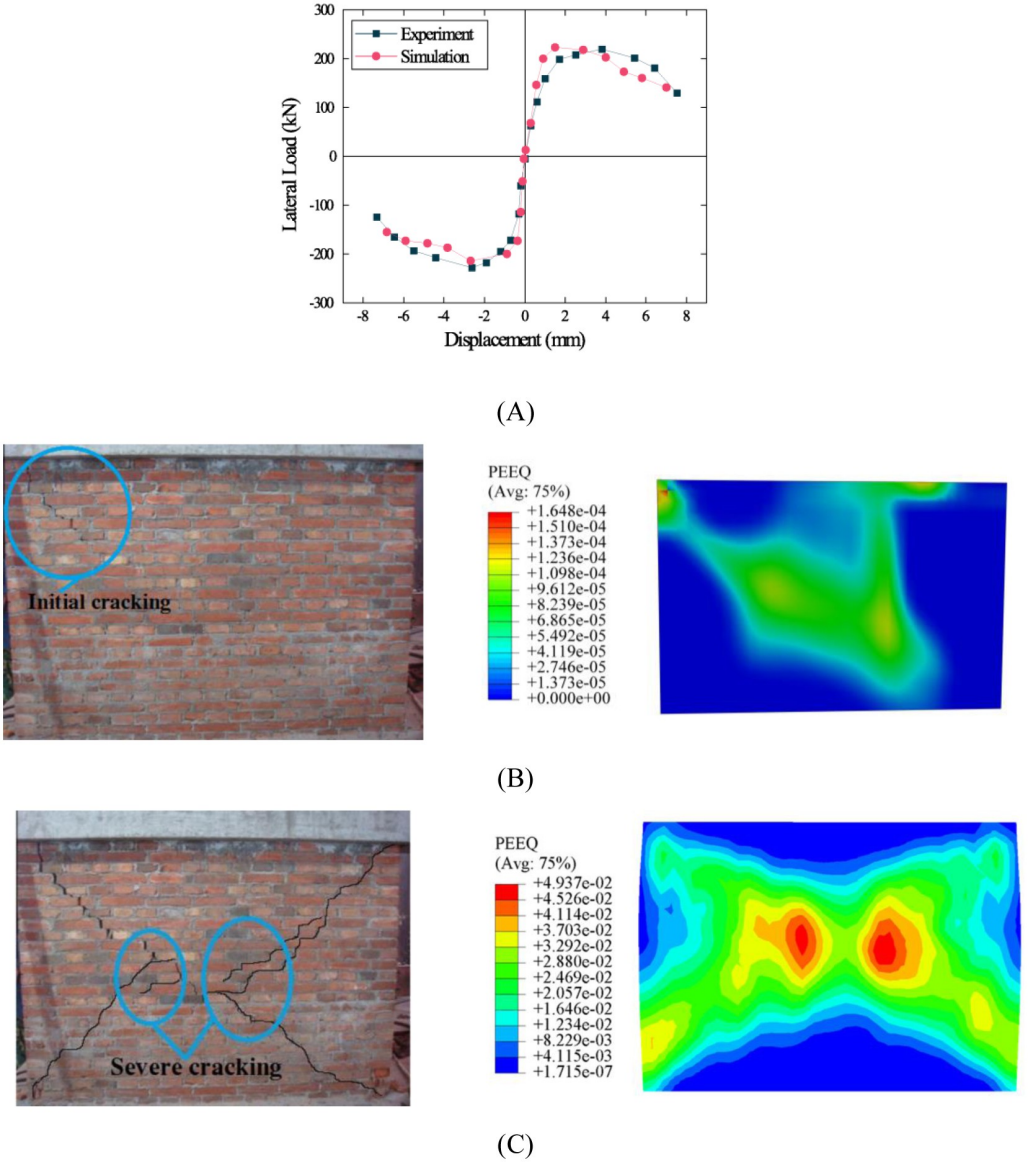
Comparison of the results of testing and simulation. (A) Force–displacement curve. (B) Initial cracking pattern and stress nephogram. (C) Severe cracking pattern and stress nephogram.

The force–displacement comparison curve obtained from the test [22] and simulation is shown in Fig 4A. In general, a similar shape of the force–displacement curves was observed through comparative analysis. Both curves showed obvious linear growth characteristics before 1.5 mm; during the period of 2–4 mm, the test curve still exhibited a gradual increasing trend, but the simulation curve showed a slow decreasing trend. When the displacement reached 7 mm, the failure load decreased to 85% of the maximum load. Although both curves displayed some differences in the range of 2–4 mm, obvious difference was observed in the maximum horizontal load between the wall and model, and the overall pattern of the force–displacement curves was consistent.

As shown in Fig 4B, the first crack was observed in the upper left corner of the wall when the cyclic horizontal load was continuously applied to the wall. With the continuous increase in lateral displacement, cracks continued to expand and enlarge, and when the displacement reached 6–7 mm, diagonal cracks along the diagonal direction of the wall formed and continued to widen, as shown in Fig 4C. Subsequently, the local mortar on the wall surface began to fall off and vertical and diagonal cracks were captured at the toe of the wall. Finally, diagonal shear cracks were formed and the test was terminated.

## Modeling program

### Model design

Through an analysis of the design dimensions of masonry walls in published articles, a summary of different aspect ratios is presented in Table 2. It can be observed from Table 2 that the aspect ratio of the masonry wall was in the range of 0.50–1.50. The size of the model in this section was based on the size of the specimen used in Section 2. Simultaneously, combined with the data usually used in the literature [49–52, 56–59], five models with different aspect ratios were designed. The aspect ratio of the model was in the range of 0.50–2.00, and the specific design parameters are displayed in Table 3.

**Table 2:** Summary of wall dimensions in different literature.

Literature	Height × Length × Width (mm) × (mm) × (mm)	Aspect ratio of masonry wall
[46]	1008 × 1500 × 240	0.67
	1008 × 2000 × 240	0.50
[49]	1100 × 1600 × 240	0.69
[50]	1560 × 1000 × 240	1.56
	1560 × 2100 × 240	0.74
[51]	1500 × 2100 × 240	0.71
[52]	1250 × 2300 × 240	0.54
[53]	1856 × 1500 × 240	1.24
[54]	1400 × 2000 × 240	0.70
[55]	2000 × 1975 × 240	1.11

**Table 3:** Design parameters of the model.

Height × Length × Width	Aspect ratio	Model number					
(mm) × (mm) × (mm)		0.2 MPa	0.3 MPa	0.4 MPa	0.5 MPa	0.6 MPa	0.7 MPa
1500 × 3000 × 240	0.50	M1-1	M1-2	M1-3	M1-4	M1-5	M1-6
1500 × 2250 × 240	0.67	M2-1	M2-2	M2-3	M2-4	M2-5	M2-6
1500 × 1500 × 240	1.00	M3-1	M3-2	M3-3	M3-4	M3-5	M3-6
2250 × 1500 × 240	1.50	M4-1	M4-2	M4-3	M4-4	M4-5	M4-6
3000 × 1500 × 240	2.00	M5-1	M5-2	M5-3	M5-4	M5-5	M5-6

### Load actions

To comprehensively explore the influence of vertical load on the seismic performance of URM masonry walls, it is extremely important to determine the value of the vertical load input in the finite element model. According to the relevant provisions of Load Code for the Design of Building Structures (GB 50009–2012) [60] in China and the explanation in this study [55], the vertical load of traditional masonry buildings is calculated by considering the live load of the floor and the self–weight of walls and floors. Generally, the vertical load is approximately 100 kPa (0.10 MPa) for each floor. Commonly used masonry buildings in China have two to seven floors [1]. Therefore, the range of vertical load input for each model with the same aspect ratio was set to 0.20–0.70 MPa.

### Constraints and interactions

In this section, the constraints adopted by the model and the interactions between interfaces are consistent with those described in Section 2.3. A completely fixed method was adopted as the boundary condition for the bottom beam. “Tie” was set between the top loading beam and masonry wall, as well as between the masonry wall and bottom beam, which could ensure that no relative slipping would exist between each part. Moreover, the bottom beam was completely fixed by setting the relevant parameters; that is, the displacement in three directions was input to zero (*u*_x_ = *u*_y_ = *u*_z_ = 0) to limit the sliding of the bottom beam. The details of the finite element model are shown in Fig 3.

## Discussion of simulation results

### Failure mode

**Fig 5:**
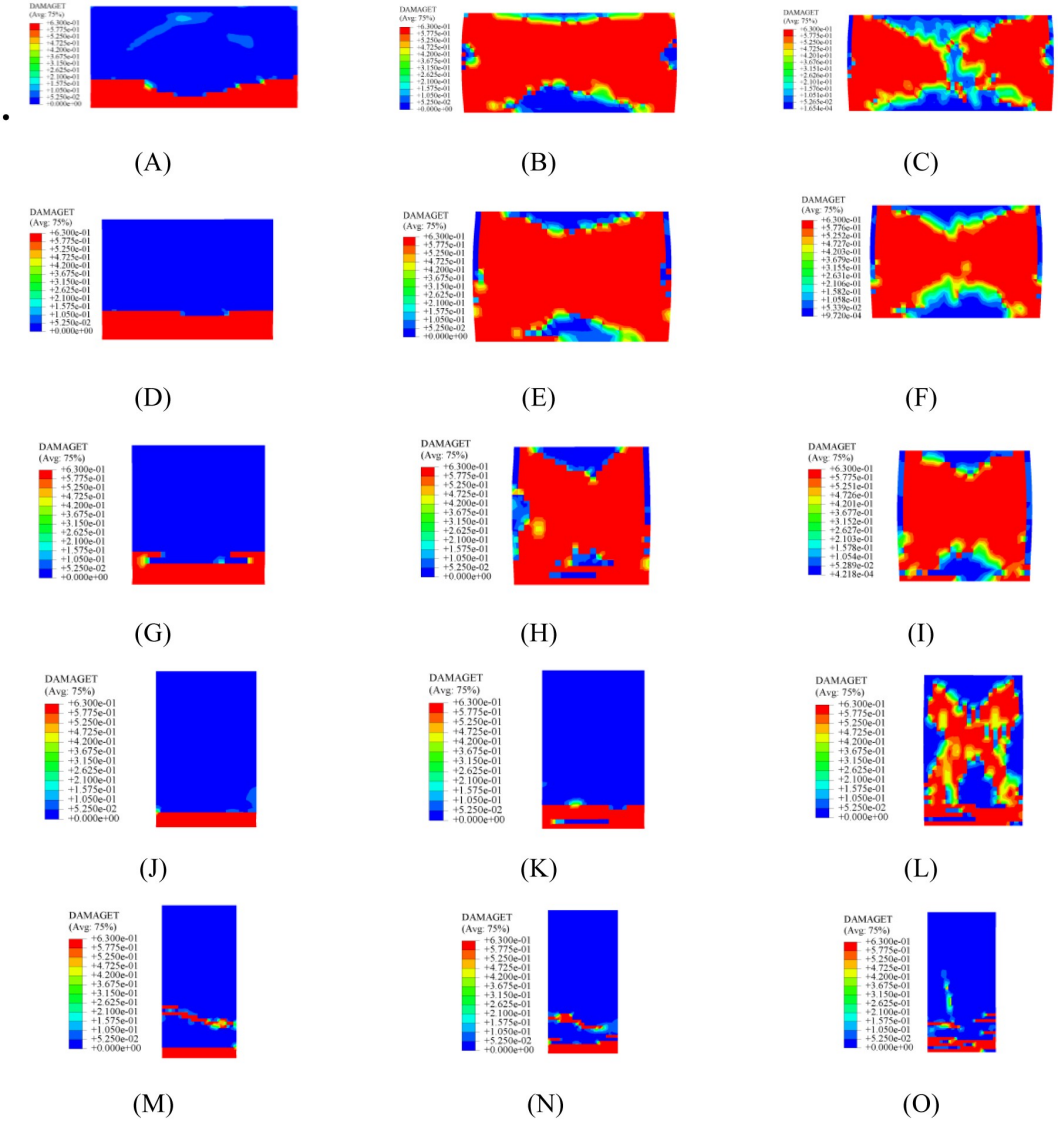
Partial stress nephogram of damage–tensile. (A) M1-1. (B) M1-3. (C) M1-5. (D) M2-1. (E) M2-3. (F) M2-5. (G) M3-1. (H) M3-3. (I) M3-5. (J) M4-1. (K) M4-3. (L) M4-5. (M) M5-1. (N) M5-3. (O) M5-5.

**Fig 6:**
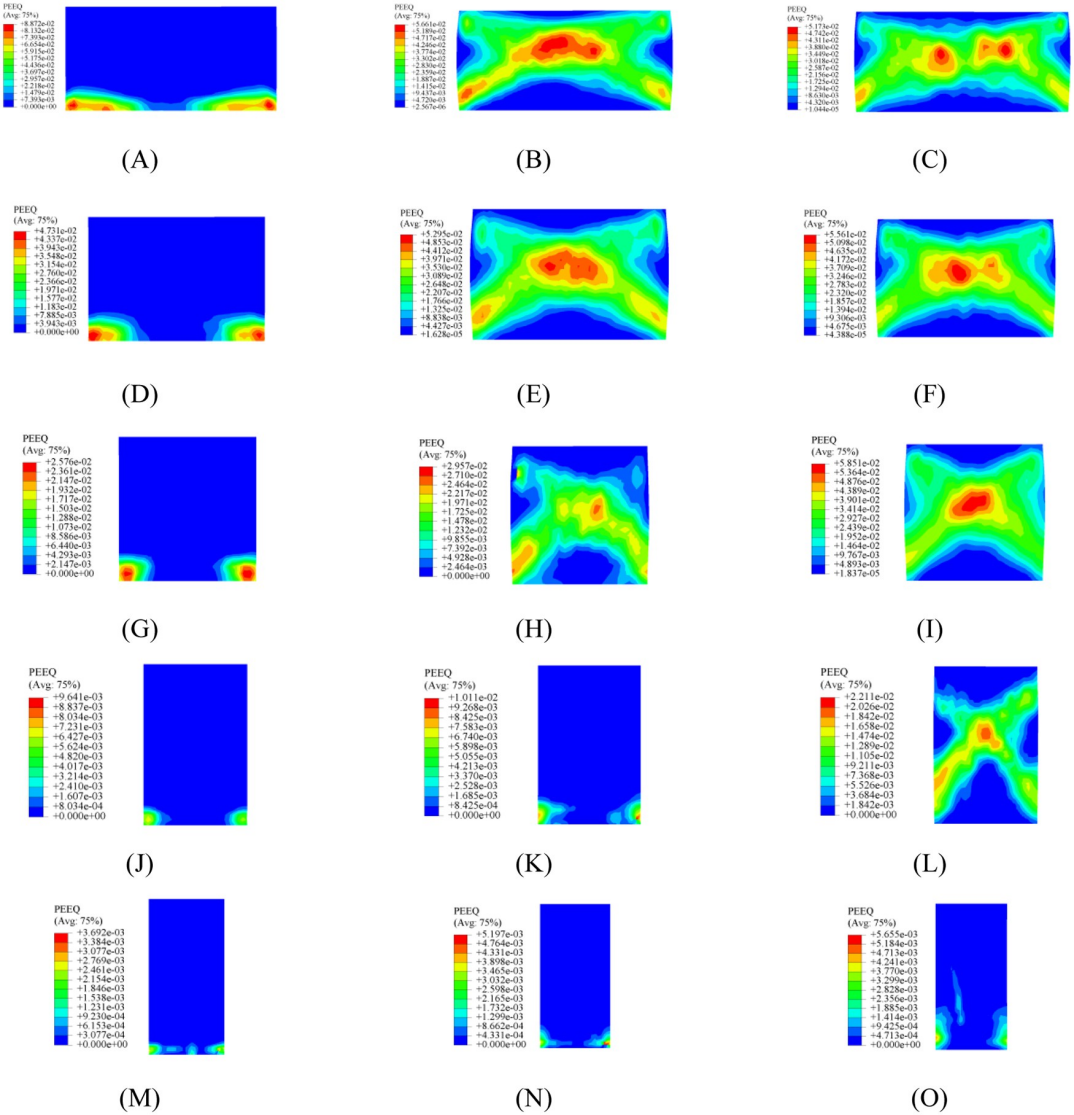
Partial stress nephogram of PEEQ. (A) M1-1. (B) M1-3. (C) M1-5. (D) M2-1. (E) M2-3. (F) M2-5. (G) M3-1. (H) M3-3. (I) M3-5. (J) M4-1. (K) M4-3. (L) M4-5. (M) M5-1. (N) M5-3. (O) M5-5.

A summary of the failure modes for all models is shown in Table 4, and the stress nephograms of damage–tensile and stress nephograms of PEEQ for partial models are presented in Figs 5 and 6, respectively. Further, the influences of the aspect ratio and vertical load on the seismic performance of the model are discussed in detail.

**Table 4:** Summary of failure modes for all models.

Aspect ratio	Vertical load (MPa)					
	0.20	0.30	0.40	0.50	0.60	0.70
0.50	F	S	S	S	S	S
0.67	F	S	S	S	S	S
1.00	F	F	S	S	S	S
1.50	F	F	F	F	S	S
2.00	F	F	F	F	F	F

^1^ Where: F represents the flexural failure mode, S represents the shear failure mode.

Through the comparison of Table 4, it can be seen that: (i) in general, two main failure modes were observed, including flexural and shear failures; (ii) when the aspect ratio was less than 1.00, the shear failure could be considered as the main failure mode of the model; (iii) flexural–shear mixed failure emerged when the aspect ratio was equal to 1.00; (iv) when the aspect ratio was greater than 1.00, the flexural failure was considered to be the main failure mode of the model; (v) when a vertical load of 0.20 MPa was applied to the model, only the flexural failure was observed, regardless of whether the aspect ratio of the model increased or decreased; (vi) the flexural shear mixed failure was captured within the range of 0.30 MPa– 0.50 MPa; (vii) the shear–dominated failure was the main failure mode of the model when a vertical load of 0.60 MPa– 0.70 MPa was applied to the model.

The possible reasons for these phenomena are as follows: (i) The "lower" wall (aspect ratio of 0.50–1.00) has more significant resistance to horizontal load than the "higher" wall (aspect ratio of 1.00–2.00), because the former has more abundant stress transfer paths (along the horizontal direction). (ii) A higher vertical load can significantly increase the normal stress between the brick element and mortar interface, which inhibits the crack from extending along the horizontal direction of the wall bottom and promotes the occurrence of shear cracks along the diagonal direction. In contrast, a lower vertical load fails to increase the normal stress between the brick element and mortar interface, which makes it easier for the wall to form horizontal cracks at the bottom and causes compression failure of the toes. By comparing the stress nephogram of damage–tensile (Fig 5), it can be found that for all models with a vertical load of 0.2 MPa, the damage was primarily concentrated on the feet of the model, and the cracks in the horizontal direction gradually emerged and expanded. For models with larger vertical loads, the damage in a large number of models was mainly characterized by diagonal cracking, and the cracks continued to widen and enlarge. However, for the M5 model, whether the vertical load increased or decreased, diagonal shear cracks failed to emerge, but the horizontal cracks at the bottom continued to increase and expand, indicating that the contribution of the vertical load to the model was no longer significant once the aspect ratio of the model exceeded a specific value.

Fig 6 shows the equivalent plastic strains of the partial models. In the Abaqus software, PEEQ is defined as the equivalent plastic strain. The main plastic strain was concentrated at the toe of the model. For the model with a smaller vertical load, the in–plane rocking response was captured and observed. One possible reason for this is that the smaller vertical load failed to limit the in–plane rotation of the model, and the strain at the toe failed to expand to the central region. In contrast, the main plastic strain appeared in the central region of the model. For the model with a larger vertical load, the rocking response of the model was limited under the action of a larger vertical load, and shear deformation was regarded as the main form of the model. Meanwhile, the equivalent plastic strain in the central region of the model was significantly higher than that in other regions, which is consistent with the test results in the relevant published literature [9, 22]. In addition, the rocking response was quite obvious because the models had a larger aspect ratio, and the influence of the vertical load on the models was considered to be negligible.

### Force–displacement curves

**Fig 7:**
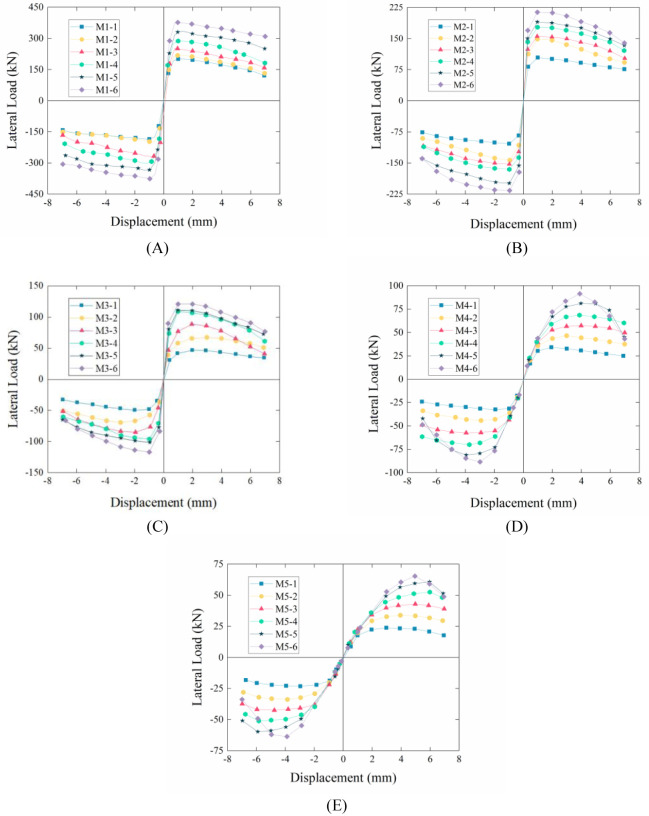
Force–displacement curves. (A) M1-1 – M1-6. (B) M2-1 – M2-6. (C) M3-1 – M3-6. (D) M4-1 – M4-6. (E) M5-1 – M5-6.

The force–displacement curves of all models are shown in Fig 7, which are drawn from the peak load and the corresponding horizontal displacement in the third-level cycle. In general, the force–displacement curve of each model was linear before the wall cracked, whether the model with the same aspect ratio but different vertical loads or the model with the specific vertical load but different aspect ratios was used. Further, the force–displacement curves began to tilt towards the displacement axis (horizontal axis), and the growth rate of the horizontal load slowed down. The curves presented a downward trend once the horizontal load reached the maximum horizontal load value.

In addition, under specific vertical load conditions, the horizontal bearing capacity of the model exhibited a decreasing regulation from Fig 7A–7E, indicating that the model with a smaller aspect ratio could resist higher horizontal loads under a specific vertical load. Under a specific aspect ratio, the horizontal bearing capacity of the model was positively correlated with the applied vertical load, indicating that a model with a larger vertical load could bear a larger horizontal load. One possible reason for this is that the friction between the brick and mortar increases under the action of a greater vertical load, and greater friction could provide greater shear resistance for the model.

However, some differences were observed between the two groups. The force–displacement curve presented a more rapid downward trend once the horizontal load exceeded the maximum load value, as shown in Fig 7A–7C, which might be caused by shear cracking and increased damage. In contrast, the force–displacement curve showed a slow downward trend after the horizontal load exceeded the maximum load value, as shown in Fig 7D and 7E, which is closely related to the rocking response of the model. The damage to the model was primarily concentrated in the lower part and hardly expanded and enlarged to the rest, which guaranteed that the model had a relatively stable post–peak horizontal load.

For all models of M1 and M2, all force–displacement curves exhibited steep rising and gradual falling stages. The corresponding horizontal load presents a regular increase with an increase in the vertical load (202 kN–383 kN for M1 and 108 kN–212 kN for M2). However, for all models–M3-M5, all force–displacement curves show a gradual rising phase and a continuous phase after the peak value. The peak load of all curves was smaller than the peak load of the M1 and M2 curves, within the range of 25 kN–130 kN. The above phenomena indicate that masonry walls can bear a higher horizontal load under the condition of a smaller aspect ratio, and the capacity of the walls to bear a horizontal load increases with a gradual increase in the vertical load.

Therefore, masonry walls with smaller aspect ratio (0.50–1.00) should be designed and constructed in practical projects. Engineering designers should avoid designing masonry walls with a larger aspect ratio (1.00–2.00). For the as-built masonry wall with a larger aspect ratio, the corresponding reinforcement measures should be taken to improve the ability of the wall to bear the horizontal load. In addition, pre-stretching measures can be implemented to improve the positive pressure on the wall along the vertical direction.

## Conclusions

The objective of this study was to investigate the effects of the aspect ratio and vertical load on the seismic performance of unreinforced masonry (URM) structures. The main conclusions are summarized as follows:

1. In Section 2, by quoting the test specimen in the published literature, comparing the results with the simulation method, and using the ABAQUS software, a macro-mechanical model was established that could accurately predict the failure mode and obtain the maximum horizontal load of URM wall.
2. The common failure modes of the URM wall model included shear and flexural failures, and flexural shear mixed failure was also observed under specific conditions.
3. Shear failure was considered as the main failure mode of the model when the aspect ratio was less than 1.00, and flexural failure was considered to be the main failure mode of the model when the aspect ratio was greater than 1.00.
4. When a vertical load of 0.20 MPa was applied to the model, only flexural failure was observed, regardless of whether the aspect ratio of the model increased or decreased. The flexural shear mixed failure was captured within the range of 0.30 MPa– 0.50 MPa, and shear failure was the main failure mode within the range of 0.60 MPa– 0.70 MPa.
5. For the model with a smaller vertical load (0.20–0.30 MPa), the damage was primarily concentrated at the bottom of the model, and the cracks in the horizontal direction gradually emerged and expanded; in contrast, for the model with a larger vertical load (0.40–0.70 MPa), the damage was primarily characterized by diagonal cracks, and the cracks further widened and enlarged.
6. For the model with a smaller vertical load (0.20–0.30 MPa), the in–plane rocking response was captured and observed, possibly because the smaller vertical load failed to limit the in–plane rotation of the model, and the strain at the toe failed to expand to the central region.
7. For the model with a larger vertical load (0.40–0.70 MPa), the damage of the model was characterized by cracks gradually forming and extending in the diagonal direction, and the equivalent plastic strain in the central region of the model was obviously higher than that in other regions, which is consistent with the test results in the published literature.
8. In general, a wall with an aspect ratio of less than 1.00 can bear a higher horizontal load, and an increase in the vertical load can significantly improve the horizontal load bearing capacity of the wall. In contrast, once the aspect ratio of the wall reaches or exceeds 1.00, the horizontal load has little effect on the increase in the horizontal load bearing capacity of the wall.
9. In practical engineering applications, to enable masonry walls to bear higher horizontal loads, it is recommended that relevant measures be taken to reduce the aspect ratio and increase the vertical load of the wall. For the as-built wall, external interference measures should be taken to strengthen the wall itself so that the wall has a higher capacity to bear a horizontal load than the original wall.
10. The numerical simulation method adopted in this study is a simple and desirable method that may be mastered and used by more researchers to further study and discuss the seismic performance of masonry structures under the action of various influencing factors.

## Supporting information

S1 Table Comparison of the results of testing and simulation. This is the data in Fig 4A.
(XLSX)

S2 Table Force–displacement curves of M1. This is the data in Fig 7A.
(XLSX)

S3 Table Force–displacement curves of M2. This is the data in Fig 7B.
(XLSX)

S4 Table Force–displacement curves of M3. This is the data in Fig 7C.
(XLSX)

S5 Table Force–displacement curves of M4. This is the data in Fig 7D.
(XLSX)

S6 Table Force–displacement curves of M5. This is the data in Fig 7E.
(XLSX)
